# Observation of spin Seebeck contribution to the transverse thermopower in Ni-Pt and MnBi-Au bulk nanocomposites

**DOI:** 10.1038/ncomms13714

**Published:** 2016-12-12

**Authors:** Stephen R. Boona, Koen Vandaele, Isabel N. Boona, David W. McComb, Joseph P. Heremans

**Affiliations:** 1 Department of Mechanical and Aerospace Engineering, The Ohio State University, Columbus, Ohio 43210, USA; 2 Department of Inorganic and Physical Chemistry, Ghent University, Gent B-9000, Belgium; 3 Center for Electron Microscopy and Analysis, The Ohio State University, Columbus, Ohio 43212, USA; 4 Department of Materials Science and Engineering, The Ohio State University, Columbus, Ohio 43210, USA; 5 Department of Physics, The Ohio State University, Columbus, Ohio 43210, USA

## Abstract

Transverse thermoelectric devices produce electric fields perpendicular to an incident heat flux. Classically, this process is driven by the Nernst effect in bulk solids, wherein a magnetic field generates a Lorentz force on thermally excited electrons. The spin Seebeck effect also produces magnetization-dependent transverse electric fields. It is traditionally observed in thin metallic films deposited on electrically insulating ferromagnets, but the films' high resistance limits thermoelectric conversion efficiency. Combining Nernst and spin Seebeck effect in bulk materials would enable devices with simultaneously large transverse thermopower and low electrical resistance. Here we demonstrate experimentally that this is possible in composites of conducting ferromagnets (Ni or MnBi) containing metallic nanoparticles with strong spin–orbit interactions (Pt or Au). These materials display positive shifts in transverse thermopower attributable to inverse spin Hall electric fields in the nanoparticles. This more than doubles the power output of the Ni-Pt materials, establishing proof of principle that the spin Seebeck effect persists in bulk nanocomposites.

## Abstract (web summary)

The spin Seebeck effect enables thermal-to-electrical energy conversion but the power generated in thin films remains low. Here, Boona *et al*. use composites of ferromagnetic conductors containing noble metal nanoparticles to show that the effect can enhance the transverse thermopower of bulk materials.

The spin Seebeck effect^1^ (SSE) offers an alternate approach to conventional thermoelectric effects for solid-state heat-to-electricity energy conversion^2^,^3^,^4^,^5^. SSE involves the injection of heat into a polarized magnetic material, resulting in a thermal spin current. This spin current is typically detected by injecting it via spin pumping^6^ into an adjacent thin film comprising a nonmagnetic metal with strong spin–orbit interactions, such as Pt. There, the inverse spin Hall effect (ISHE)^7^ produces a transverse electric field proportional to the polarization and density of the incident spin current. These parameters are controlled experimentally by applying a magnetic field to alter the magnetic polarization, and by adjusting the temperature drop across the structure to alter the spin current density.

The geometry of SSE structures is identical to that in which the anomalous Nernst effect (ANE) is observed, except ANE occurs entirely within single-phase conducting ferromagnets. This is why metallic ferromagnets are avoided in experiments designed to isolate and understand the physics of SSE. However, combining SSE and ANE is a useful engineering strategy for enhancing the total transverse thermopower *S*_*xyz*_ in ferromagnetic heterostructures without the need for large applied magnetic fields. Direct implementation of this concept for energy conversion means utilizing devices where the electrically active regions are thin films, leading to a different set of problems: although thin films allow for the design of devices with mechanical flexibility^2^, their small cross-sectional area and inherently large electrical resistance limits the maximum possible power output of such devices to the mW level at most.

Here we demonstrate that SSE and ANE can be combined in an alternate bulk geometry better suited for energy conversion. Devices based on this concept hold potential for producing power at the W to kW level, as they comprise bulk materials that can be manufactured using large-scale production processes similar to those already in use for conventional thermoelectric devices. In this geometry, the entire device volume is both electrically and thermally active, enabling significantly lower source impedance and potentially higher voltage output compared with thin-film structures. Proof of concept is demonstrated here in a Ni-Pt composite, where the addition of Pt nanoparticles results in a significant enhancement of the transverse thermopower because of combined contributions from both the Nernst effect (*S*_Nernst_) and SSE (*S*_SSE_). Together with low electrical resistivity *ρ*, the transverse power factor PF_*xyz*_=*S*_*xyz*_^2^/*ρ* in the composites is increased 2–5 times compared with the reference sample, and the transverse thermoelectric figure of merit *zT*_*xyz*_=*T*·PF_*xyz*_/*κ* (*κ* being the thermal conductivity and *T* the absolute temperature) increases by an order of magnitude. This result establishes that not only can SSE be observed in bulk nanocomposites, but it can also be utilized to produce significantly more electrical power from the same temperature gradient relative to single-phase magnetic materials.

## Results

### The spin Seebeck effect in thin-film structures

**Figure 1:**
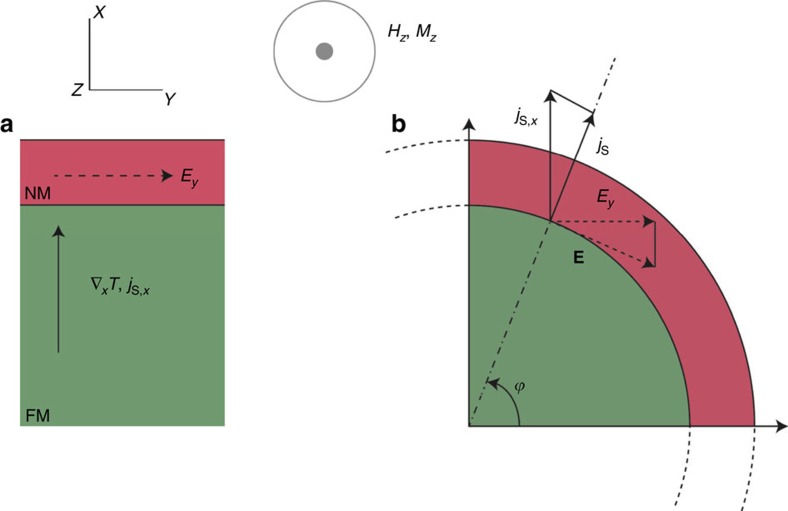
Schematic representation of the spin Seebeck effect in different geometries. Spatial directions (*x*,*y*,*z*) are specified by the legend at the top. The concentric circles indicate the applied magnetic field *H*_*z*_ and magnetization *M*_*z*_ for both panels are pointing out of plane. (**a**) Geometrical layout of a conventional spin-Seebeck effect (SSE) geometry showing a normal metal (NM) film (red) on top of a ferromagnetic insulator (FMI, green). A spin current *j*_S*,x*_ is driven by a temperature gradient ∇_*x*_*T* within the FM. The polarization direction of *j*_S*,x*_ is determined by *H*_*z*_ and *M*_*z*_. This spin current is injected into the NM, where the inverse spin Hall effect (ISHE) produces an electric field *E*_*y*_. (**b**) One quadrant of the same structure folded upon itself in an FMI (green)/NM (red) core–shell configuration. Under the same conditions as (**a**) (*H*_*z*_, *M*_*z*_,∇_*x*_*T*), an ISHE field **E** again arises in the NM, but now its directional components must be taken into account. Integrating **E** over the full circle shows that *E*_*x*_ cancels out, but non-zero *E*_*y*_ remains (see equation (2)). This very general idea can be applied to a variety of arbitrary interface geometries, thereby providing the conceptual basis for SSE in random bulk composites.

A typical thin-film longitudinal SSE geometry is depicted schematically in Fig. 1a. A temperature gradient ∇_*x*_*T* is applied to a ferromagnetic insulator (FMI) possessing magnetization *M*_*z*_ set by applied field *H*_*z*_. ∇_*x*_*T* generates a magnon flux that carries heat^8^ and spin along *x*. At the surface these magnons impinge on an adjacent normal metal (NM) film whose thickness is comparable to its spin diffusion length *l*_s_, typically 1–10 nm. This spectrally dependent^9^,^10^ spin pumping action polarizes free electrons in the NM, resulting in a perpendicular electric field *E*_*y*_ via ISHE. The magnitude of *E*_*y*_ depends on the polarization and magnitude of the injected spin current. This figure makes it apparent that from the perspective of energy conversion, electrical detection of thermally generated spin currents makes SSE functionally equivalent to a transverse thermopower.

From Fig. 1a, we define the SSE coefficient 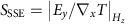. Typical values of *S*_SSE_ in Y_3_Fe_5_O_12_(YIG)/Pt heterostructures are ∼0.1 μV K^−1^ at 300 K. Recent work on Fe_3_O_4_/Pt multilayer thin films^11^, YIG/NiO/Pt heterostructures^12^ and spin flopped antiferromagnets^13^ show coefficients near 6 μV K^−1^ at various temperatures. These values are much lower than the longitudinal thermopower of classical thermoelectric devices (200 μV K^−1^ in tetradymites^14^), so the intrinsic figure of merit in thin-film SSE heterostructures^4^,^15^ is many orders of magnitude lower than in traditional semiconductor-based thermoelectrics.

As spin is not conserved, the spin diffusion length of spin-polarized electrons in the NM (*l*_s_) and magnons in the FMI (*L*_s_) limits the geometry, size and materials suitable for any SSE-based structures. For example, ISHE produces an electric field **E**_ISHE_ in the NM only if the NM thickness *t* is approximately equal to *l*_s_ (that is, when only 0.001 vol% or 10 p.p.m. of the total device volume is electrically active). If *t* is much greater than *l*_s_, then **E**_ISHE_ is short circuited by electrons in the region of NM where spin current has decayed. Second, *L*_s_ limits the active thermal volume of FMI from which spin is injected into the NM. A conservative estimate for *L*_s_ in YIG at 300 K is 10 μm (refs 16, 17, 18), although some experiments suggest a length scale of ∼250 nm (refs 19, 20) is relevant to SSE. Regardless, this length is significantly shorter than the macroscopic sample and substrate thickness *L* that is typically 0.5 mm. As ∇_*x*_*T* in *S*_SSE_ is determined by Δ*T*_*x*_/*L* and not Δ*T*_*x*_/*L*_S_, only 1% of Δ*T*_*x*_ participates in generating *E*_*y*_; although thermal excitation produces larger spin current densities than resonant or electrical methods^21^, only a small fraction of the thermal energy is actually converted into electrical energy.

Pure SSE experiments require that the ferromagnetic material be insulating so that spin is transported only by magnons and not free electrons. Otherwise, **E**_ISHE_ in the NM would mix with ordinary and anomalous Nernst thermopower from the ferromagnet to give a total transverse thermopower *S*_*xyz*_=*S*_Nernst_+*S*_SSE_ (ref. 22). Careful separation of Nernst and SSE in bilayer structures is necessary when studying detailed origins of physical mechanisms^23^. For energy conversion, however, it is advantageous to combine Nernst and SSE into a single transverse thermopower, as long as both effects have the same polarity. This maximizes the voltage output per temperature gradient and enables lower electrical resistance, resulting in larger power output.

Multilayer structures are one way to accomplish this: adding additional thermally and electrically active layers^11^,^24^,^25^ can increase *S*_*xyz*_ to 1.8 μV K^−1^ and the extrinsic power factor *S*_*xyz*_^2^/*R* (*R* being the film sheet resistance) to 2 pW K^−2^. Though these values can be reached at room temperature and with little to no applied magnetic field, the additional layers are still thin films. This means the electrically and thermally active regions of the device remain a small fraction of the total volume (much less than 1 vol%), and hence the devices have relatively high electrical impedance, and a significant portion of thermal energy goes unused. As an alternative approach, here we propose bulk composites as a scalable and robust way to make the entire device volume both electrically and thermally active, thus providing a viable pathway towards higher and more efficient power output.

### The SSE in bulk materials

Figure 1b illustrates the conceptual basis for SSE in a bulk composite geometry. In general, **E**_ISHE_=*ρ* tan*θ*_SH_ (**j**_S_ × ***σ***), where *ρ* and *θ*_SH_ are the electrical resistivity and spin Hall angle of the NM, respectively, whereas **j**_s_ represents the spin current density in the NM, and **σ** indicates the spin current polarization. Experimentally, **j**_s_ is essentially proportional to **∇***T*, and **σ** is determined by the ferromagnet magnetization tensor **M** that is set by an external applied magnetic field **H**. As discussed above, **E**_ISHE_ is partially short circuited by electrical conduction within the NM, resulting in attenuation that scales with exp[−*t*/*l*_s_]. Combining these and all other prefactors into a proportionality factor *A*, we have


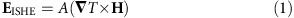


To justify the composite geometry, we consider a spherical FMI particle coated with a NM film (Fig. 1b). When subjected to **H**=(0,0,*H*_*z*_) and **∇***T*=(∇_*x*_*T*,0,0), equation 1 indicates **E**_ISHE_ develops tangentially to the NM/FMI interface in the (*x,y*) plane. Integrating **E**_ISHE_ over the entire sphere reveals *E*_*x*_=0 whereas *E*_*y*_ (*ϕ*)=*A*|∇*T*||**H**|sin^2^*ϕ*, such that *E*_*y*_≥0 for all *ϕ*. Assuming the sphere's radius *r*>>*t*, then *S*_*xyz*_ becomes:


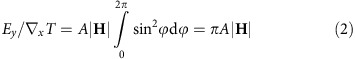


Equation (2) gives a value of *E*_*y*_ that is one-half that of a slab with the same length as the periphery of the sphere. Admittedly, attenuation of **E**_ISHE_ via short circuit through the NM is slightly enhanced in this spherical geometry over the traditional planar geometry, but we numerically estimate this factor to be ∼15% for *t ≈l*_s_.

By equation (2), we propose that any bulk composite material comprising an FMI surrounded by a percolated network of sufficiently small NM grains should display a prominent *S*_*xyz*_=*S*_SSE_. Yet, this geometry is not the most favourable option for demonstrating such an effect, for two reasons. First, this approach requires compaction and sintering of FMI particles coated with thin shells of NM material. This presents processing difficulties, as a percolated nanoscale conducting pathway of NM grains must be maintained after sintering to observe SSE and extract electrical current. Second, spin transfer from FMIs into NMs depends on the FMI/NM interface quality^26^ that is difficult to control in bulk materials.

We address both issues through the inverted geometry, wherein NM nanoparticles (NMNPs) are embedded within a ferromagnetic conductor (FMC) matrix. By the same logic described above, individual NMNPs in contact with FMC grains will display within them nonzero **E**_ISHE_, so long as the depth of NMNPs along *x* is comparable to *l*_s_. Current can then be extracted through the FMC matrix, and **E**_ISHE_ in the NMNP (*S*_SSE_) will be superimposed on the background Nernst signal of the FMC (*S*_Nernst_), as in ref. 22. This approach circumvents the need for a percolated conducting path of NM, enables the NMNPs to add their **E**_ISHE_ to the transverse voltage (if the polarities are chosen right) and simultaneously decreases the voltage source impedance. The latter effects both improve power efficiency.

This approach furthermore enables improved interfacial spin transfer efficiency, as spin currents entering the NMNPs arise from both magnons and spin-polarized electrons. However, the magnitude of **E**_ISHE_ is still limited by the degree of spin polarization in the FMC and the frequency of electronic spin-flip scattering at the FMC/NM boundaries. The sign of **E**_ISHE_ within the NM depends on the sign of *θ*_SH_ and the relative orientations of **∇***T* and **M**, and hence we nominally expect the *S*_SSE_ polarity to be independent of *S*_Nernst_. In addition, the electrical conductances of the FMC and the NM must be impedance matched to have the appropriate additive effect on the ISHE and Nernst voltages that are electrically connected like voltage sources in parallel; that is, the FMC cannot have high electrical conductance.

### Experimental results

Details of the synthesis, measurement, characterization and data analysis are provided in the Methods and Supplementary Information.

We synthesized five samples, summarized in Table 1. The first sample consisted of pure polycrystalline Ni nanoparticles (NiNP) produced via precipitation reaction. This sample was used to establish a baseline for *S*_Nernst_ in the matrix material. Two other samples were NiNP-PtNP composites made via coprecipitation from solution of Ni and 2 wt% Pt. The fourth and fifth samples were made with MnBi as the host material and Au nanoparticles (AuNP) as the embedded NM phase.

In order to mitigate agglomeration of the PtNP and prevent possible alloying of Ni with Pt, the Ni-based composite samples were sintered at 250 °C, well below the melting point of the constituent phases. This resulted in samples that were mechanically stable but only ∼50% dense, with no lattice parameter shifts or other signs of alloying (see Supplementary Fig. 1 and Supplementary Notes).

**Figure 2:**
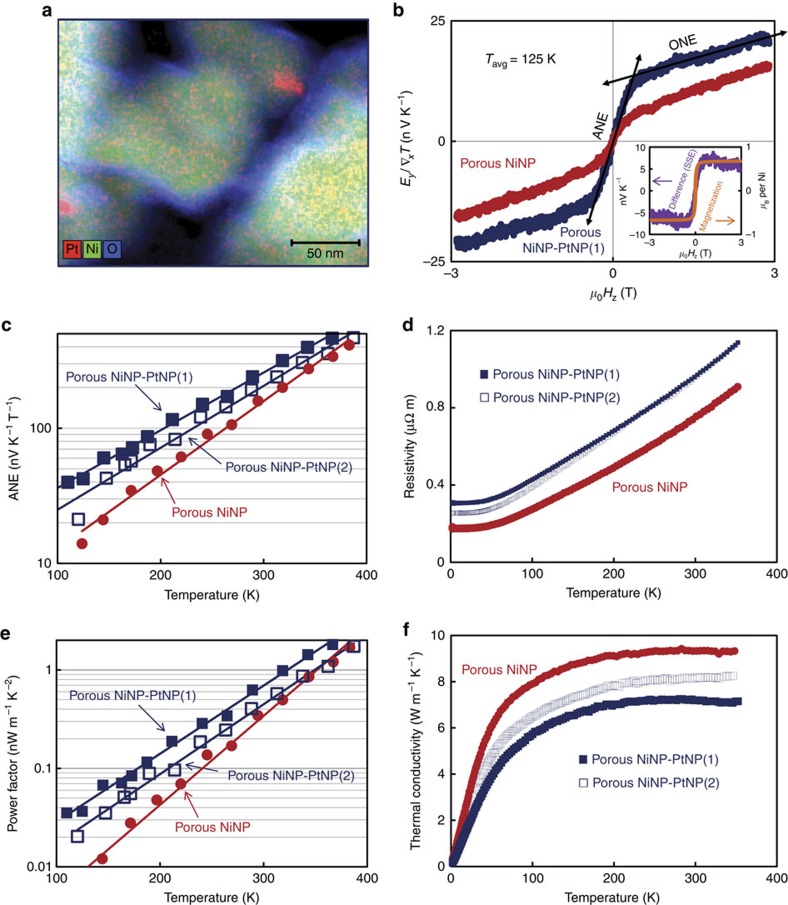
Microstructure and transport properties of Ni-Pt composites. (**a**) High-angle annular dark-field (HAADF) image of NiNP-PtNP(2) overlaid with false-colour elemental mapping of Pt (red), Ni (green) and O (blue) determined by X-ray energy-dispersive spectroscopy (XEDS) in a scanning transmission electron microscope (STEM). (**b**) Transverse thermopower versus applied field at 125 K for porous NiNP (red) and porous NiNP-PtNP(1) (blue). The slopes at low and high fields give the ANE and ONE coefficients, respectively. The inset shows the difference between the red and blue curves (purple), along with the sample magnetization (orange). (**c**) ANE coefficients of the three samples versus temperature. Solid lines are a guide for the eye. (**d**) Resistivity of all three samples versus temperature. (**e**) Temperature dependence of the intrinsic power factor of NiNP (red circles), NiNP-PtNP(1) (solid blue squares) and NiNP-PtNP(2) (empty blue squares) at *H*_*z*_=0.1 T. Solid lines are a guide for the eye. (**f**) Thermal conductivity of all three samples versus temperature.

Figure 2a shows a representative electron micrograph of a composite sample obtained after sintering. From this image, we see that the sample consists of Ni particles ∼100 nm in diameter and partially coated in a thin layer of NiO ∼5 nm thick. These Ni particles also appear to be decorated with small Pt nanoparticles ∼5 nm in size (see Supplementary Figs 2 and 3 and Supplementary Notes for additional images). The PtNP sometimes form larger agglomerates nearly 20 nm in size, as seen in parts of the image. These particles, including the agglomerates, are well within the size range where we expect an appreciable ISHE field to arise within the Pt because of thermal spin injection from the adjacent Ni.

The thin layer of NiO observed at the edges of the Ni grains implies that the transport properties of the samples we report here are likely much different from those of pure, dense, bulk Ni. This emphasizes the importance of utilizing a reference sample prepared in the same manner as the composites. As it remains unclear how many interfaces in the sample are Ni/Pt and how many are Ni/NiO/Pt, we must consider what happens in the latter case. The presence of thin layers of electrically insulating NiO is certain to affect charge and spin transport between the Ni and Pt particles. However, as NiO is antiferromagnetic, spin currents can still propagate through thin layers of this material; this has been demonstrated in several recent studies, one of which observed spin currents transmitted through NiO layers up to 100 nm thick in Y_3_Fe_5_O_12_/NiO/Pt heterostructures, as well as multiple studies that suggest the addition of NiO actually enhances the transmission of spin currents under certain conditions^12^,^27^,^28^,^29^. The presence of NiO undoubtedly has a detrimental effect on the electrical resistivity and power factor of both the reference and composite materials, although we anticipate these effects may be partially negated by a corresponding decrease in thermal conductivity.

With this in mind, we examine the transverse thermopower data collected at 125 K and shown in Fig. 2b for the NiNP reference and NiNP-PtNP(1). The inset shows the difference in thermopower between the two samples overlaid with the NiNP magnetization at the same temperature. From the main figure, we see that the slopes of the transverse thermopowers versus field are approximately equal at fields >0.5 T, where the magnetization is saturated (the ordinary Nernst effect (ONE)), whereas they are significantly different at fields <0.5 T, where the magnetization is changing (the ANE). The difference between the thermopowers of the two materials tracks the NiNP magnetization, consistent with the hypothesis that an ISHE field arises within the PtNP. We note that essentially no hysteresis was observed in the thermopower, and very minimal hysteresis was detected in the NiNP magnetization.

The ANE coefficients [*E*_*y*_*/*∇_*x*_*T*]*/H*_*z*_ (that is, low-field thermopower slopes) for each of the NiNP-based samples are plotted versus temperature in Fig. 2c. At every temperature, the samples containing PtNP have larger ANE coefficients than the sample without, indicating an additional magnetization-dependent contribution to the thermopower. A plot of the high-field ONE coefficients versus temperature is included in Supplementary Fig. 4. We do not observe any appreciable difference in ONE coefficient among the three samples, indicating that the addition of a few wt% PtNP has no significant or systematic effect on the transverse thermopower when the magnetization is saturated.

The electrical resistivity of all three samples is shown in Fig. 2d. The samples containing PtNP are very similar to one another and slightly more resistive than the reference sample that is ∼10 times more resistive than dense Ni. The resistivity and ANE data are combined in Fig. 2e, where we plot the intrinsic transverse power factor PF_*xyz*_=(*S*_*xyz*_)^2^*/ρ* at various temperatures for all three samples. As there is essentially no remnant magnetization in these materials, PF_*xyz*_ was calculated using the transverse thermopower at *H*_*z*_=0.1 T, an applied magnetic field easily reached with permanent magnets. These data show that the addition of PtNP increases PF_*xyz*_ between 2 and 5 times compared with the matrix material alone. Thermal conductivity (*κ*) data are also presented in Fig. 2f. Nanostructuring dominates *κ* in these samples, as indicated by the relatively low magnitude and extremely flat temperature dependence. Like *ρ*, *κ* is reduced by ∼20% between the NiNP and the NiNP-PtNP sample 1; however, the variation in *κ* between the two Pt-containing samples is larger than the corresponding change in *ρ*, presumably because of variations in microstructure resulting from slight differences in processing conditions.

These data can be combined to calculate a transverse thermoelectric figure of merit *zT*_*xyz*_=*T*·PF_*xyz*_/*κ*, where *T* is the absolute temperature. The transverse *zT*_*xyz*_ values (not shown) follow the trend in PF_*xyz*_ amplified by their variation in *κ*. The *zT*_*xyz*_ values remain small, as they are in all conventional metals and SSE structures. Regardless, we observe an order of magnitude increase in *zT*_*xyz*_ at 100 K between the composite and reference samples, a dramatic improvement that can be attributed entirely to the spin Seebeck effect.

To provide a further check on the composite concept, we examined two additional samples where the host material (MnBi) has an ANE coefficient with polarity opposite to that of Ni, but the NMNPs (Au) still have positive spin Hall angles. Au was used instead of Pt because it has a much longer spin diffusion length, allowing for relatively large Au particles (above 50 nm) to be present without completely short circuiting the ISHE electric field. The wet chemistry used to prepare Ni and Pt NP's cannot be applied in this case because MnBi is moisture sensitive, and hence a different approach was utilized (see Methods).

**Figure 3:**
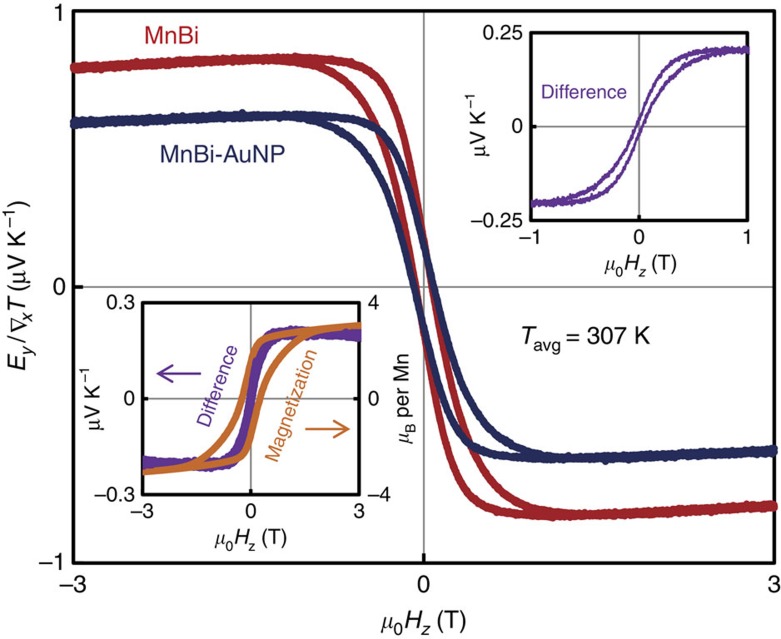
Transverse thermopower of MnBi-Au composites at 307 K. The red trace indicates the thermopower of MnBi, whereas that of MnBi-AuNP is shown in blue. The lower left inset shows the difference between these curves (purple) as well as the magnetization of the MnBi at 307 K (orange), whereas the upper right inset emphasizes the hysteresis loop in the difference plot.

The transverse thermopower of pure MnBi and a MnBi-AuNP composite at 300 K are depicted in Fig. 3. To our knowledge, these are the first published data of the thermomagnetic properties of MnBi. The presence of AuNP in the composite shifts *E*_*y*_*/*∇_*x*_*T* towards a more positive value, consistent with positive *θ*_SH_ in Au, and therefore with the existence of an ISHE contribution from the AuNP. The difference between the transverse thermopowers of the two materials is plotted in the lower left inset, along with the magnetization of MnBi powder.

Admittedly, any shift in transverse thermopower where the magnitude decreases is difficult to uniquely attribute to an ISHE field in the NMNP, as this may also result from simpler mechanisms like short circuits of the ANE electric field across larger NM particles. However, in this case, we observe the difference curve follows the same general field dependence of the magnetization, including a small hysteresis loop (upper right inset of Fig. 3), indicating that the shift in thermopower is not simply a constant offset arising from a short circuit of the ANE field. The size of this hysteresis loop is not particularly meaningful, as (unlike thin-film samples) we do not expect the ISHE field to follow exactly the hysteresis loop of the entire sample; instead, the ISHE hysteresis loop should reflect the magnetization of individual MnBi particles from which spin currents are thermally injected into neighbouring AuNP. The absolute magnitude of the difference (the SSE voltage) appears to be significantly larger in this sample than it is in the Ni-Pt composites, although the relative change compared with the reference sample is similar. This is probably a reflection of differences in the ratio of electrical resistivity between the FMC and NM phases; Pt and Ni are roughly within a factor of two of each other, whereas our MnBi sample is at least an order of magnitude more resistive than Au (see Supplementary Fig. 5). In this regard, the FMC matrix phase is less likely to short circuit the SSE voltage in MnBi-AuNP than in NiNP-PtNP, leading to larger *S*_SSE_.

## Discussion

Put together, these data demonstrate that the SSE can be observed and exploited not only in thin-film structures, but now also in bulk composite materials. Though we discuss our results above in terms of the conventional thermoelectric power factor concept, which we parse into intrinsic and extrinsic when comparing composites with thin films, it is perhaps more intuitive to simply consider the total power output of hypothetical devices based on each concept. As bulk composites allow for electrical power to be extracted through their entire volume and thus provide far less internal resistance than thin films, such devices would be capable of producing significantly more total power from the same temperature gradient.

Broadly speaking, the composite concept applies to a wide variety of material combinations, and it invites further experimental and theoretical optimization of spin transport and spin pumping in irregular and/or random geometries. Further developments in materials selection and processing may lead to substantial increases in thermoelectric performance of magnetic composites beyond the order of magnitude improvement we report here, including large remnant transverse thermopowers that can produce voltage even in the absence of applied magnetic fields. If so, this approach may enable the development of low-cost SSE-based devices that require no vacuum processing and have dimensions like those of conventional thermoelectric materials, providing access to high power thermal energy conversion applications.

## Methods

### Material synthesis

All samples used for transport property measurements were polycrystals compacted from powders by spark plasma sintering into 10 mm diameter cylindrical pellets ∼1–2 mm thick. The nanoparticles used in this study were either Au (AuNP), which had diameters before sintering of ∼50 nm and were purchased commercially (Sigma Aldrich), or Pt (PtNP), which had diameters before sintering of 2–5 nm and were chemically and mechanically dispersed among Ni nanoparticles during synthesis at the Ohio State University.

PtNP and NiNP were synthesized by reducing Ni(NO_3_)_2_.6H_2_O with NaBH_4_ in the presence of H_2_PtCl_6_.6H_2_O and sodium citrate as complexing agent. The resulting NiNP were amorphous (a-NiNP) and intimately mixed with PtNP, as observed by imaging in the scanning transmission electron microscope (STEM). The a-NiNP was then converted to crystalline NiNP by post reduction treatment with N_2_H_4_.H_2_O. Hydrogen hexachloroplatinate(IV) hydrate, ca. 40% Pt, and sodium borohydride powder 99% were purchased from Acros Organics. Sodium citrate dihydrate >99%, nickel nitrate hexahydrate 99.99%, 64–65% hydrazine monohydrate and 99.8% ethylene glycol were supplied by Sigma Aldrich. All chemicals were used as received.

In a typical synthesis, 4 g Ni(NO_3_)_2_.6H_2_O salt and 0.0428, g H_2_PtCl_6_.6H_2_O were dissolved in 250 ml water and stabilized by 8.77 g sodium citrate. Subsequently, the solution was heated to 80 °C and 1.56 g NaBH_4_ was dissolved in 20 ml water. Then, 7.15 g NaOH was added and the mixture was stirred vigorously for 15 min. Next, 10.8 ml N_2_H_4_.H_2_O in 20 ml ethyleneglycol was added to the solution and stirred for 1 h at 80 °C. The nanocomposite powder was collected magnetically and washed thoroughly with water and ethanol. The dried powder was then compacted by spark plasma sintering under 50 MPa of uniaxial pressure at 250 °C held for 30 min in a graphite die under continuous vacuum.

Polycrystalline powders of MnBi were synthesized through a combination of arc melting, grinding, sieving and annealing, using the methods similar to those described in ref. 30. The MnBi-AuNP composite sample was created by mixing 5 vol% AuNP with 95 vol% MnBi powder (−125 mesh) and sonicating in a hexane bath at 35 kHz for 20 min. As MnBi is moderately air and water sensitive, hexane was used to prevent decomposition. The suspension was dried then sintered under 50 MPa of uniaxial pressure at a temperature of 200 °C held for 5 min in a graphite die under continuous vacuum. The control sample of MnBi was synthesized from the same batch of powder under the same conditions but without AuNP.

### Structural and compositional characterization

X-ray diffraction analysis was performed using a Rigaku MiniFlex Diffractometer with Cu-K_*α*_ radiation. Based on the X-ray diffraction results, we successfully synthesized high-purity polycrystalline MnBi and MnBi-AuNP materials containing no discernible impurity phases (see Supplementary Fig. 6 and Supplementary Notes). We also observe no discernible peak shift in the Ni-Pt composites, indicating no alloying occurred between Ni and Pt during sintering at 250 °C.

Microstructural analysis was performed on several samples of control and composite materials by scanning electron microscopy (SEM) and STEM with high-angle annular dark-field (HAADF) imaging and X-ray energy-dispersive spectroscopy (XEDS). The HAADF-STEM imaging and XEDS spectrum collection was performed using a monochromated FEI Titan 60-300 STEM equipped with a Super-X XEDS collection system. The SEM work and XEDS spectra were collected using the FEI Sirion field emission SEM equipped with the EDAX Octane Super SDD system.

SEM analysis of the AuNP shows Au inclusions ranging in size from ∼50 nm particles up to 20 μm agglomerates. STEM and XEDS analysis of the NiNP-PtNP before sintering indicated 2–5 nm PtNP. After sintering, we observed various nanostructured features that included 20 nm Pt agglomerates and what appear to be PtNP under 5 nm in diameter widely dispersed and decorating the Ni grains. Additional representative images are provided in the Supplementary Information.

### Measurements

Parallelepiped pieces of each sample were cut using a rotary diamond saw. Silver epoxy (EpoTech) was used as a conducting adhesive to attach gold-coated copper leads, heat spreaders and heat sinks to the samples in a five-probe configuration. All transport measurements were performed in a Quantum Design Physical Property Measurement System via the Thermal Transport Option. Transverse thermopower measurements were performed using the five-probe technique with a home-built breakout box, whereas electrical resistivity was measured using a conventional AC four-probe method. Seebeck coefficient and thermal conductivity were determined using a continuous measurement protocol.

For transverse thermopower measurements, a steady-state temperature difference Δ*T*_*x*_ was established and then the corresponding transverse voltage Δ*V*_*y*_ was recorded while continuously sweeping the applied magnetic field *H*_*z*_ back and forth between maximum and minimum values of *H*_*z*_=30 kOe at a constant rate of 50 Oe s^−1^. The data were analyzed by averaging out the odd components to isolate the hysteresis curve for each temperature point. These signals were then normalized by the corresponding field-dependent temperature difference as well as by the associated lengths in order to arrive at the intrinsic *S*_*xyz*_ coefficient.

### Data availability

The data that support the findings of this study are available from the corresponding author upon request.

## Additional information

**How to cite this article:** Boona, S. R. *et al*. Observation of spin Seebeck contribution to the transverse thermopower in Ni-Pt and MnBi-Au bulk nanocomposites. *Nat. Commun.* **7**: 13714 doi: 10.1038/ncomms13714 (2016).

**Publisher's note**: Springer Nature remains neutral with regard to jurisdictional claims in published maps and institutional affiliations.

## Supplementary Material

Supplementary Information Supplementary Figures 1-6 and Supplementary Discussion

## Figures and Tables

**Table 1:** Summary of the samples studied here.

**Sample name**	**Host compound**	**Nanoparticles**	**NP size (nm)**	ANE of host*	***θ***_***SH***_ **of NP**
NiNP	Ni	Ni	100	+	N/A
NiNP-PtNP(1)	Ni	Ni+Pt	100 (Ni), 5 (Pt)	+	+
NiNP-PtNP(2)	Ni	Ni+Pt	100 (Ni), 5 (Pt)	+	+
MnBi	MnBi	N/A	N/A	−	N/A
MnBi-AuNP	MnBi	Au	50 nm	−	+

^*^Based on the spin Seebeck sign convention that is opposite to that of Gerlach.
