# Supplementary material for: Observation of spin Seebeck contribution to the transverse thermopower in Ni-Pt and MnBi-Au bulk nanocomposites
Source: Nat Commun. 2016 Dec 12;7:13714. doi: 10.1038/ncomms13714 (PMC5159888; doi:10.1038/ncomms13714)
Supplement: Supplementary Information — Supplementary Figures 1-6 and Supplementary Discussion [file ncomms13714-s1.pdf]

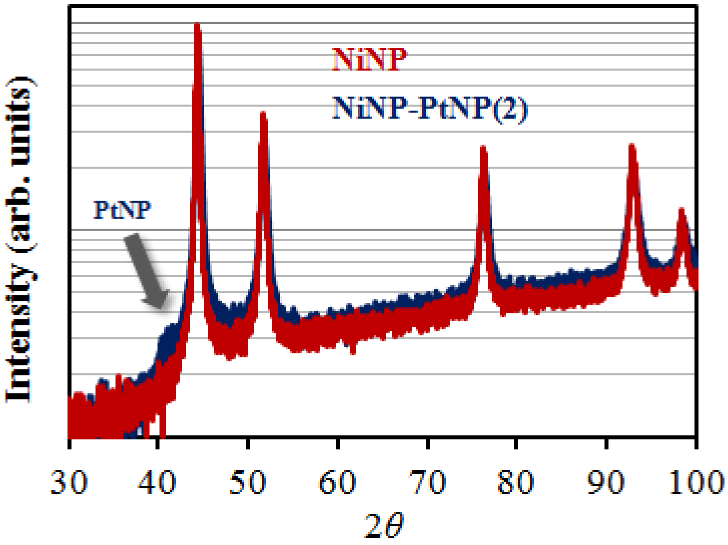

Supplementary Figure 1. Powder x-ray diffractometry of sintered NiNP and NiNP-PtNP(2).

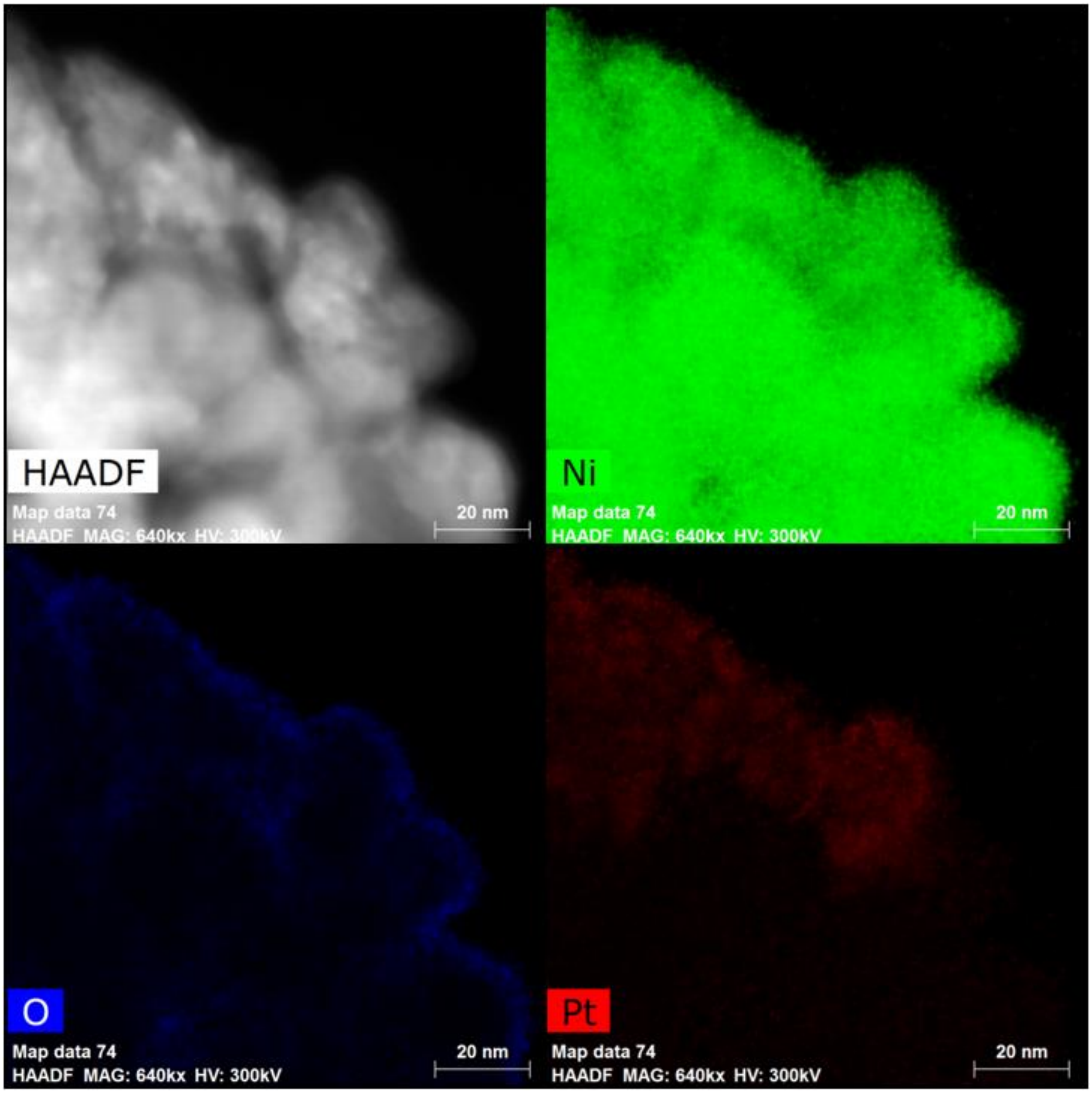

Supplementary Figure 2. HAADF-STEM image and XEDS elemental maps of NiNP-PtNP(2). HAADF-STEM (top left) image is displayed, along with elemental maps of Ni (green, top right), O (blue, bottom left), and Pt (red, bottom right).

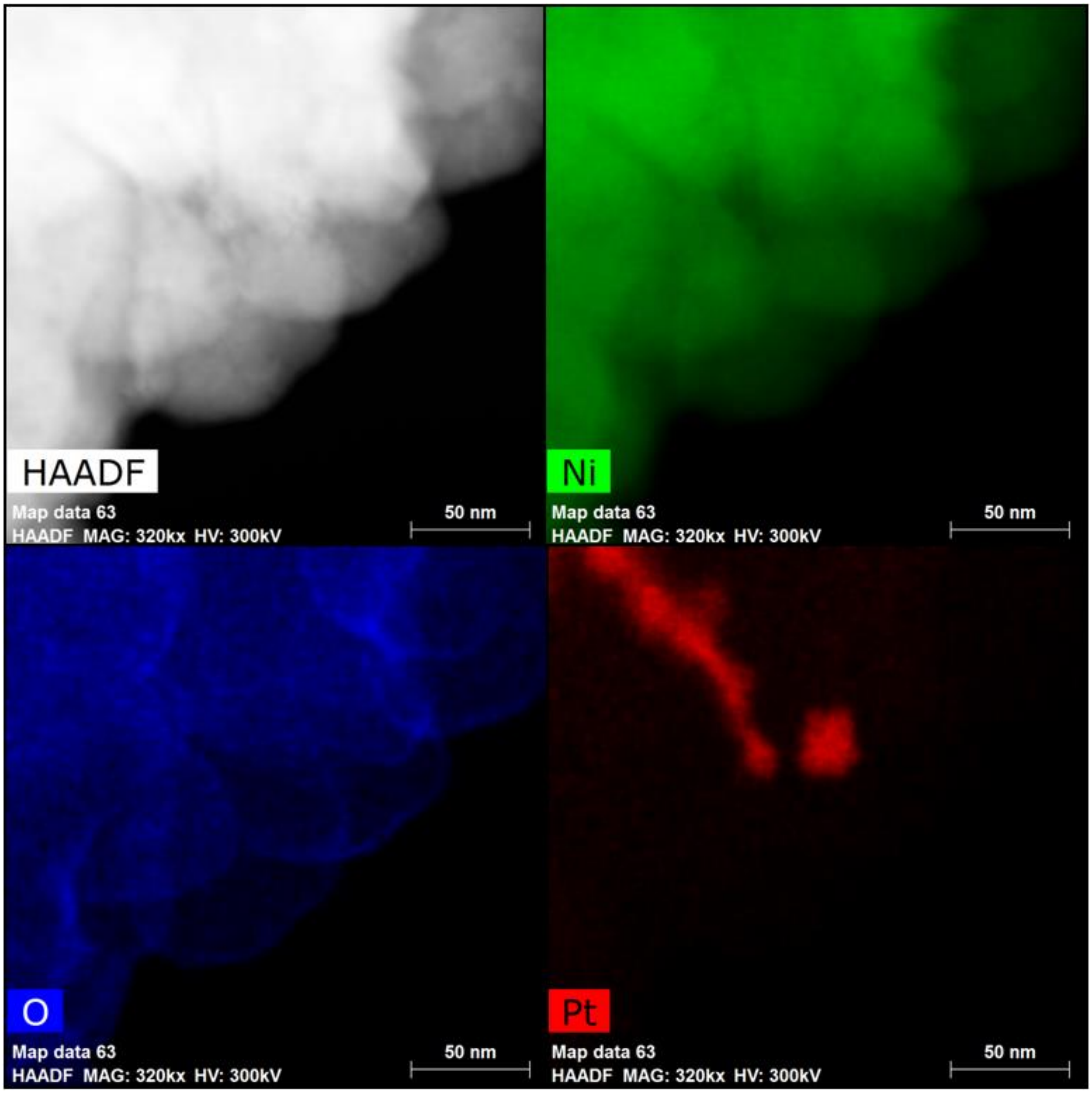

Supplementary Figure 3. Additional HAADF-STEM XEDS elemental maps of NiNP-PtNP(2). HAADF-STEM (top left) image is displayed, along with elemental maps of Ni (green, top right), O (blue, bottom left), and Pt (red, bottom right).

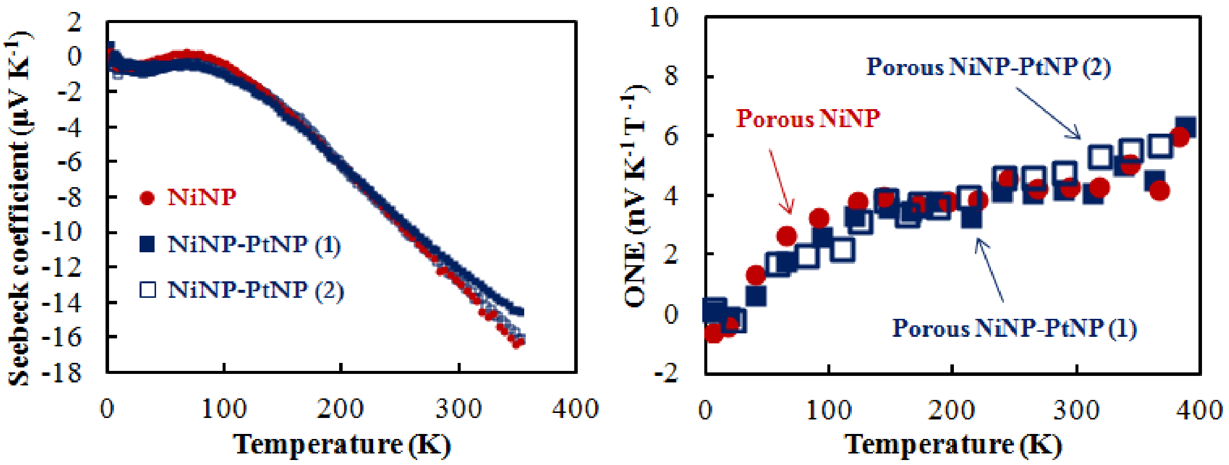

Supplementary Figure 4. Seebeck coefficient (left panel) and ordinary Nernst coefficient (right panel) of the three Ni-based samples.

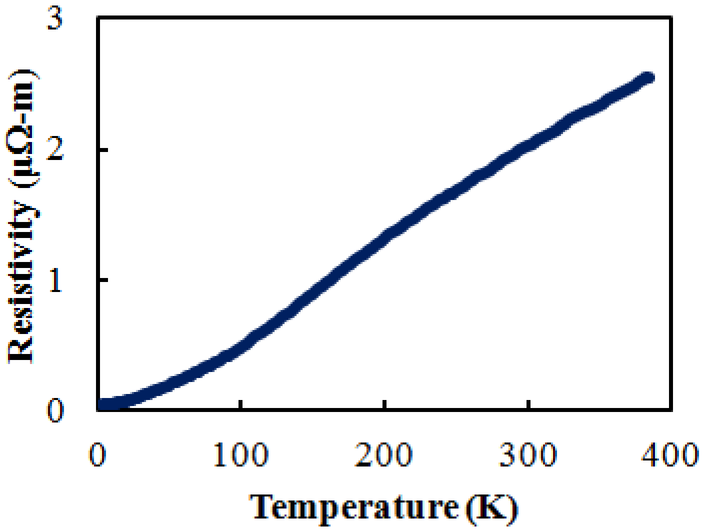

Supplementary Figure 5. Electrical resistivity vs temperature for polycrystalline MnBi.

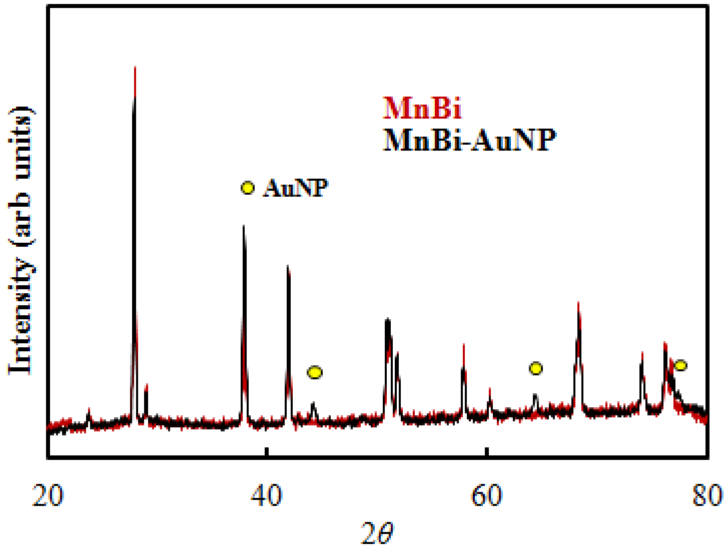

Supplementary Figure 6. Powder XRD of sintered MnBi and MnBi-AuNP. No trace of a phase other than MnBi is seen, but the detection limit of secondary phases in XRD spectra is limited to a few %.

Supplementary Discussion

Electron microscopy

Supplementary Figure 2 shows a second representative HAADF-STEM image (first image is in the main text) of the NiNP-PtNP(2) sample with associated false-colored elemental maps of Ni, Pt, and O. This image shows what appear to be Ni particles less than 50nm in size coated in NiO, with a portion of the image coated in a thick layer of Pt particles and/or agglomerates ~20 nm in size.

A third representative HAADF-STEM image of NiNP-PtNP(2) is shown in Supplementary Figure 3, along with the associated false-colored elemental maps of Ni, Pt, and O. This image shows what appear to Ni particles less than 100nm in size coated in NiO and decorated with Pt particles. A concentrated vein of Pt particles and/or agglomerates ~10-20 nm thick is visible in the upper center/left of the image.

X-ray diffractometry

Supplementary Figure 1 shows the powder XRD results obtained for the sintered NiNP and NiNP-PtNP(2) samples. We observe no noticeable shift in lattice parameter after sintering, indicating no detectable alloying of Pt and Ni. Instead, near $2\theta = 40°$ we see the primary diffraction peak of pure Pt metal. All peaks are clearly broadened relative to their intensity, indicating sub-100 nm coherent scattering volumes.

Supplementary Figure 6 shows the powder x-ray diffraction (XRD) results obtained for the sintered MnBi and MnBi-AuNP samples made from the same batch of MnBi powder. As seen in the figure, we obtain high purity MnBi with no discernible impurity peaks visible in

either sample. The only difference between the control and composite samples are the additional peaks of Au present in the latter. Similar to what is seen in Supplementary Figure 1, the Au peaks are clearly broadened relative to their intensity, indicative of sub-100nm coherent scattering volumes.
